# Association between six different types of anthropometric indices and arterial stiffness measured by brachial-ankle pulse wave velocity in hypertensive Chinese adults

**DOI:** 10.1016/j.heliyon.2024.e28523

**Published:** 2024-03-27

**Authors:** Feng Hu, Wei Zhou, Tao Wang, Chao Yu, Lingjuan Zhu, Huihui Bao, Xiaoshu Cheng

**Affiliations:** a Department of Cardiovascular Medicine, the Second Affiliated Hospital of Nanchang University, Nanchang, Jiangxi, 330006, China; b Department of Cardiology, Fujian Medical University Union Hospital, Fuzhou, 350000, China; c Jiangxi Provincial Cardiovascular Disease Clinical Medical Research Center, Nanchang, Jiangxi, 330006, China; d Jiangxi Sub-center of National Clinical Research Center for Cardiovascular Diseases, Nanchang, Jiangxi, 330006, China; e Center for Prevention and Treatment of Cardiovascular Diseases, the Second Affiliated Hospital of Nanchang University, Nanchang, Jiangxi, China

**Keywords:** body mass index, Waist-hip ratio, Waist-to-height ratio, A body shape index, Body round index, Pulse wave velocity

## Abstract

**Background:**

The associations of body fat parameters with arterial stiffness measured by brachial-ankle pulse wave velocity in hypertensive patients were scarce.

**Methods:**

This cross-sectional study analyzed 4322 hypertensive adults. The correlations between the anthropometric indexes (body mass index [BMI], waist circumference, waist-tohip ratio [WHR], waist-to-height ratio [WHtR], a body shape index [ABSI], body round index [BRI]) and ba-PWV values were analyzed using multivariable linear regression model.

**Results:**

In both sex categories, linear regression models showed that BMI levels were inversely related to baPWV (adjusted-*β* per SD increase in male: −0.51, 95% CI -0.66 to −0.36, P < 0.001; female: −0.50, 95% CI -0.63 to −0.37, P < 0.001). Waist circumference positively correlated with baPWV only in male hypertensive individuals. BaPWV positively correlated to WHR or WHtR levels (adjusted-*β* per SD increase: 0.32, 95% CI 0.21 to 0.43, P < 0.001; 0.64, 95% CI 0.47 to 0.82, P < 0.001; respectively), ABSI (adjusted-*β* per SD increase for ABSI × 100: 0.27, 95% CI 0.18 to 0.36, P < 0.001) and BRI (adjusted-*β* per SD increase: 0.64, 95% CI 0.46 to 0.81, P < 0.001) levels. The relationship between anthropometric indices and arterial stiffness based on baPWV values were also consistent. ABSI had the highest predictive power of arterial stiffness (area under the curve, 0.594; P < 0.001).

**Conclusion:**

In Chinese adults with hypertension, BMI was inversely related to baPWV, while WHR, WHtR, ABSI and BRI were positively related. Waist circumference positively correlated with baPWV only in male hypertensive individuals.

## 1 Introduction

Since the 1980s, obesity has steadily increased worldwide, reaching pandemic proportions [1]. The obesity epidemic poses one of the greatest threats to public health because it substantially facilitates incident cardiovascular risk factors, such as hypertension, dyslipidemia, diabetes and obstructive sleep apnoea [2]. Obesity also causes the development of cardiovascular disease (CVD) and related mortality independently of other risk factors [[3], [4], [5]]. In consequence, identifying practical anthropometric indices to diagnose obesity is important in clinical practice. In spite of the fact that body mass index (BMI) is widely used as a measure of obesity, it does not differentiate muscle from fat mass, nor does it reflect fat distribution, nor does it take sex differences into account when it comes to fat content [3,4,6].

There appears to be a better way to measure visceral obesity than BMI by measuring waist circumference, waist-to-hip ratio (WHR) and waist-to-height ratio (WHtR) and the latter are often more strongly correlated with cardiovascular risk factors and CVD mortality [3,7]. However, the high correlation of waist circumference, WHR, WHtR with BMI restricted their usefulness [3,[8], [9], [10], [11], [12]]. To overcome defects of these classical obesity metrics, some new anthropometric indices were proposed. Krakauer et al. [11] proposed a body shape index (ABSI) based on height and BMI, which reflects risk of death better than BMI and waist circumference alone [9,10,[13], [14], [15]]. In addition, body roundness index (BRI), combined waist circumference and height together in one algorithm to evaluate health status [9,10,15].

It has been suggested that cardiovascular dysfunction is one of the factors underlying obesity-related CV events [16]. Various stress conditions, including hypertension, hyperglycemia, and dyslipidemia, lead to a gradual stiffening of the arterial wall over time [17]. Arterial stiffness contributes to the progression of CVD because of vascular damage. As a measure of central arterial stiffness, increasing carotid-femoral pulse wave velocity (cfPWV) was associated with CVD in well-functioning general adults [18] and hypertensive patients [19]. An instrument that is more convenient and repeatable to evaluate arterial stiffness is the brachial-ankle wave velocity (baPWV), which is highly associated with the cfPWV and exhibits similar characteristics to the aortic PWV [[20], [21], [22]]. Therefore, baPWV could also be viewed as a surrogate marker of CVD [20,21,[23], [24], [25]], especially suitable for large-scale population-based study [22].

The prevalence of hypertension among adults in China is about 23.2%, and obesity is commonly associated with hypertension [26]. According to the results of aortic PWV, arterial stiffness is associated with a 48% increased risk of major cardiovascular events at first onset [27]. Among hypertensive individuals, arterial stiffness could influence risk of CV events because it plays a crucial role in hypertension pathogenesis [28]. The relationship between obesity indices and arterial stiffness has been shown in past studies, but there have been questions about the validity of this finding [[29], [30], [31], [32], [33], [34], [35], [36], [37], [38], [39], [40]]. Compared to subcutaneous fat, visceral fat is more strongly associated with CV events [41,42].

We are unaware of any previous studies investigating the relationship between multiple classical or new obesity indices and arterial stiffness in hypertensive patients based on baPWV. Using BMI, WC, WHR, DHR, ABSI, and BRI, this study examined the relationships between mean baPWV and body fat parameters in Chinese adults with hypertension.

## 2 Patients and methods

In accordance with the Declaration of Helsinki, the study was approved by the Ethics Committee of the Institute of Biomedicine, Anhui Medical University (No. CH1059). We obtained written informed consent from all subjects.

### 2.1 Study design and participants

Participants came from the ongoing China H-type Hypertension Registry Study (ChiCTR1800017274). Prior reports have discussed the methods and criteria used to collect data and determine inclusion and exclusion [[43], [44], [45]]. In a nutshell, this is a real-life, multicenter observational study conducted in Wuyuan of China from March 2018. A survey was conducted to assess control rates for hypertension and CVD risk factors [[43], [44], [45]]. Of the eligible participants, 5233 finished the measurement of baPWV. A total of 4322 patients were included in our study after excluding two subjects without a BMI, 78 individuals with ankle brachial index less than 0.9 [9,25], 104 individuals with atrial fibrillation, 334 individuals with coronary heart disease (CHD), 343 individuals with stroke, and 50 individuals with heart failure.

### 2.2 Data collection

An interdisciplinary team of cardiologists, public health physicians, and volunteers collected the data, including questionnaire survey, anthropometric indices, laboratory assay and auxiliary examinations. Demographic characteristics (age, sex), lifestyle habits (smoking and drinking), medical history [diabetes mellitus (DM), dyslipidemia and atrial fibrillation etc.) and medication usage were included in the questionnaire. The Omron HBP-1300 Professional Portable Blood Pressure Monitor was used to measure the participant's systolic blood pressure (SBP) and diastolic blood pressure (DBP). The SBP and DBP were calculated as the average of the last three readings after a 5-min rest period. An electrocardiogram (ECG8322) and a history of atrial fibrillation were used to detect atrial fibrillation.

### 2.3 Anthropometric measurements

A number of anthropometric indices were measured, including weight, height, waist circumference, and hipline. Our measurements were taken using an inelastic measuring tape to determine height, waist circumference, and hipline. Calculation of BMI is based on the kilograms per meter squared divided by the kilograms per meter squared. We calculated the WHR and WHtR by dividing the waist circumference in centimeters by the hipline and height in centimeters.

The following formulas were used to calculate ABSI and BRI [[9], [10], [11], [12], [13], [14], [15],[46], [47], [48]]:

$$\text{ABSI}=\frac{\text{Waist}\;\text{circumference}}{\left({\text{BMI}}^{\frac{2}{3}}\times {\text{Height}}^{\frac{1}{2}}\right)}$$

$$\text{BRI}=364.2-365.5\times \sqrt{1-\frac{{\left(\frac{\text{Waist}\;\text{circumference}}{2\pi }\right)}^{2}}{{\left(0.5\times \text{Height}\right)}^{2}}}$$

### 2.4 Biochemical markers

After fasting ovenight, blood samples were collected by venipuncture. A clinical laboratory for kidney disease at the National Clinical Research Center (Guangzhou, China) processed all venous samples [[43], [44], [45]]. A variety of biomarkers [serum total homocysteine, fasting blood glucose (FBG), total cholesterol (TC), total triglyceride (TG), high-density lipoprotein cholesterol (HDL-C), low-density lipoprotein cholesterol (LDL-C), uric acid and creatinine, blood urea nitrogen (BUN), total and direct bilirubin, aspartate aminotransferase (AST) and alanine aminotransferase (ALT)] were measured using automatic clinical analyzers (Beckman Coulter AU680) [[43], [44], [45]]. Diabetes was defined in our study as FBG greater than 7.0 mmoL/l, or self-reported diabetes. A person with dyslipidemia showed one or more of the following features: elevated TG (≥2.3 mmol/L), elevated TC (≥6.2 mmol/L), elevated LDL-C (≥4.1 mmol/L) and reduced HDL-C (<1.0 mmol/L) or on appropriate lipid-lowering medication [49]. Chronic Kidney Disease Epidemiology Collaboration (CKD-EPI) equation was used to estimate glomerular filtration rate (eGFR) [43].

### 2.5 BaPWV measurements

The measuring method of baPWV and ABI using a BP-203RPEIII analyzer (Omron Health Care, Kyoto, Japan) have been described previously [[43], [44], [45]]. The participants fasted, did not take cardiovascular medication, and sat in a supine position for at least 5 min before receiving this measurement. In both brachial and tibial arteries, pressure waveforms were recorded using semiconductor pressure sensors to measure the transmission time between initial rises. Using the height of the sampling points, we estimated the distance between baPWV. Based on the formula (La-Lb)/Tba, we calculated the ba-PWV value [[43], [44], [45]]. Analysis was based on the highest value of left or right sides baPWV [[43], [44], [45]]. While baPWV value has been shown to have prognostic significance, with a threshold of increased risk that exist around 18 m/s [[43], [44], [45]]. Therefore, baPWV≥18 m/s was considered as increased arterial stiffness in our analysis.

### 2.6 Statistical analysis

There were two types of variables presented: continuous variables with mean ± standard deviation/median (quartiles) and categorical variables with frequencies (percent). In order to compare data characteristics, the chi-square test was used for categorical variables, and the Student's T test or Mann Whitney *U* test was used for continuous variables depends on whether it's normally distributed.

Multivariable linear regression models were used to estimate the beta coefficients (β) of different anthropometric indices levels on the baPWV values. Confounders were not adjusted for in the crude model. Age, sex, mean blood pressure (MAP), and heart rate (HR) were adjusted in the model I. The model Ⅱ was confounder model. We selected covariates on the basis that, when added to this model, it changed the matched effect size by at least 10 percent (Supplementary Table 1). The confounder model was considered the main model. Furthermore, the median value of each anthropometric index was entered as a continuous variable in the models to test for linear trends. Using penalized spline regression (a fitted smoothing curve) and the generalized additive model, the dose-response relationship of anthropometric indices with baPWV levels was investigated.

The analyses were conducted using R (http://www.rproject.org, version 4.1.2) and Empower 2.0 (R) (http://www.empowerstats.com). It is considered statistically significant only if the *P*-value is greater than 0.05, which is two-tailed.

## 3 Results

### 3.1 Patient characteristics

In this study, 5, 049 hypertensive adults were analyzed (age: 64.15 ± 9.54 years; 49.31 percent were male). The quartile of baPWV presented participants’ characteristics in Table 1. Because ABSI was too small, ABSI × 100 was represented. Age, MAP, SBP, DBP, HR, ABSI, homocysteine, FBG, TC, HDL-C, creatinine, BUN, prevalence of DM, usage rate of hypoglycemic agents tended to increase with baPWV. BMI, waist circumference, BRI, eGFR, ALT, proportion of males and smoker tended to decrease with mean baPWV (all P values < 0.05, Table 1). Supplementary Table 2 also presented the characteristics of participants grouped by sex.

**Table 1:** Clinical characteristics of participants grouped by the baPWV quartiles.

Characteristics	Total	baPWV quartiles (m/s)				*P-value*
		Q1 [9.67, 15.31]	Q2 [15.31, 17.46]	Q3 [17.47, 20.05]	Q4 [20.05, 46.45]	
Number of subjects (n)	4322	1080	1080	1081	1081	
Age (years)	64.15 ± 9.54	58.36 ± 8.97	62.66 ± 8.52	65.85 ± 8.28	69.71 ± 8.56	<0.001
Male, n (%)	2131 (49.31%)	597 (55.28%)	535 (49.54%)	516 (47.73%)	483 (44.68%)	<0.001
SBP (mmHg)	147.25 ± 17.49	137.31 ± 14.07	143.97 ± 14.65	150.50 ± 16.05	157.21 ± 18.31	<0.001
DBP (mmHg)	89.00 ± 10.87	88.12 ± 9.73	88.06 ± 10.44	88.86 ± 11.15	90.97 ± 11.79	<0.001
MAP (mmHg)	108.42 ± 11.38	104.51 ± 9.87	106.70 ± 10.33	109.41 ± 11.08	113.05 ± 12.26	<0.001
HR (times/min)	75.81 ± 14.39	72.57 ± 12.46	73.94 ± 12.37	76.66 ± 13.88	80.08 ± 17.16	<0.001
Height (m)	1.56 ± 0.08	1.58 ± 0.08	1.56 ± 0.08	1.55 ± 0.08	1.54 ± 0.08	<0.001
Weight (Kg)	56.93 ± 10.72	60.97 ± 10.66	57.41 ± 10.31	56.19 ± 10.33	53.15 ± 10.08	<0.001
BMI (kg/m^^2^)	23.36 ± 3.52	24.24 ± 3.50	23.45 ± 3.48	23.28 ± 3.50	22.47 ± 3.37	<0.001
Waist circumference (m)	0.82 ± 0.10	0.84 ± 0.09	0.82 ± 0.10	0.82 ± 0.10	0.81 ± 0.09	<0.001
Hipline (m)	0.91 ± 0.07	0.93 ± 0.07	0.92 ± 0.08	0.91 ± 0.07	0.90 ± 0.06	<0.001
WHtR	0.53 ± 0.06	0.53 ± 0.06	0.53 ± 0.06	0.53 ± 0.06	0.53 ± 0.06	0.333
WHR	0.90 ± 0.07	0.90 ± 0.07	0.89 ± 0.07	0.90 ± 0.07	0.90 ± 0.08	0.131
ABSI*100	8.09 ± 0.48	7.99 ± 0.46	8.05 ± 0.46	8.11 ± 0.47	8.22 ± 0.51	<0.001
BRI	3.99 ± 1.23	4.02 ± 1.20	3.95 ± 1.24	4.02 ± 1.26	3.96 ± 1.22	0.358
Smoking status, n (%)						0.001
Never	2329 (53.89%)	573 (53.06%)	546 (50.56%)	599 (55.41%)	611 (56.52%)	
Former smoker	724 (16.75%)	166 (15.37%)	175 (16.20%)	189 (17.48%)	194 (17.95%)	
Current smoker	1269 (29.36%)	341 (31.57%)	359 (33.24%)	293 (27.10%)	276 (25.53%)	
Drinking status, n (%)						0.020
Never	2746 (63.54%)	652 (60.37%)	680 (62.96%)	702 (64.94%)	712 (65.86%)	
Former drinker	447 (10.34%)	105 (9.72%)	109 (10.09%)	122 (11.29%)	111 (10.27%)	
Current drinker	1129 (26.12%)	323 (29.91%)	291 (26.94%)	257 (23.77%)	258 (23.87%)	
Homocysteine (μmol/L)	15.02 (12.47–19.43)	14.20 (12.21–18.30)	14.77 (12.39–18.81)	15.07 (12.46–19.32)	16.18 (13.10–20.88)	0.003
FBG (mmol/L)	6.13 ± 1.63	5.82 ± 1.00	6.11 ± 1.77	6.23 ± 1.72	6.36 ± 1.82	<0.001
TC (mmol/L)	5.15 ± 1.10	5.07 ± 1.07	5.11 ± 1.11	5.20 ± 1.10	5.23 ± 1.13	0.001
TG (mmol/L)	1.78 ± 1.32	1.74 ± 1.26	1.78 ± 1.32	1.81 ± 1.48	1.79 ± 1.20	0.719
HDL-C (mmol/L)	1.50 ± 0.40	1.47 ± 0.38	1.49 ± 0.40	1.51 ± 0.40	1.52 ± 0.41	0.011
LDL-C (mmol/L)	2.94 ± 0.79	2.92 ± 0.77	2.92 ± 0.78	2.97 ± 0.79	2.95 ± 0.81	0.366
Uric acid (mmol/L)	430.52 ± 121.36	436.54 ± 125.91	429.62 ± 125.24	429.26 ± 117.83	426.66 ± 116.09	0.269
Creatinine (mmol/L)	67.00 (56.00–82.00)	67.00 (56.00–80.00)	65.00 (55.00–80.00)	68.00 (55.00–82.00)	69.00 (56.00–86.00)	0.012
BUN (mmol/L)	5.41 ± 1.79	5.31 ± 1.71	5.36 ± 1.64	5.43 ± 1.88	5.53 ± 1.92	0.030
eGFR (ml/min/1.73 m^2)	86.88 ± 19.41	92.33 ± 18.12	88.97 ± 18.48	85.35 ± 19.31	80.86 ± 19.81	<0.001
Total bilirubin (mmol/L)	14.38 ± 6.40	14.51 ± 6.35	14.16 ± 6.03	14.41 ± 6.48	14.45 ± 6.75	0.589
Direct bilirubin (mmol/L)	5.35 ± 2.04	5.37 ± 1.99	5.29 ± 1.95	5.35 ± 2.07	5.38 ± 2.16	0.687
AST (U/L)	24.00 (20.00–30.00)	24.00 (20.00–31.00)	24.00 (20.00–30.00)	24.00 (20.00–29.00)	24.00 (20.00–30.00)	0.599
ALT (U/L)	17.00 (12.00–24.00)	18.00 (13.00–26.00)	17.00 (13.00–25.00)	16.00 (12.00–23.00)	16.00 (12.00–23.00)	<0.001
DM, n (%)	768 (17.77%)	131 (12.13%)	181 (16.76%)	220 (20.35%)	236 (21.83%)	<0.001
Dyslipidemia, n (%)	1567 (36.26%)	381 (35.28%)	394 (36.48%)	393 (36.36%)	399 (36.91%)	0.879
Antihypertensive drugs, n (%)	2535 (58.65%)	660 (61.11%)	608 (56.30%)	611 (56.52%)	656 (60.68%)	0.029
Hypoglycemic agents, n (%)	170 (3.93%)	24 (2.22%)	37 (3.43%)	57 (5.27%)	52 (4.81%)	<0.001
Lipid-lowering agents, n (%)	78 (1.80%)	22 (2.04%)	21 (1.94%)	23 (2.13%)	12 (1.11%)	0.259
Antiplatelet agents, n (%)	72 (1.67%)	14 (1.30%)	17 (1.57%)	22 (2.04%)	19 (1.76%)	0.591
Ankle brachial index	1.15 ± 0.08	1.14 ± 0.08	1.16 ± 0.08	1.16 ± 0.08	1.15 ± 0.08	<0.001
baPWV (m/s)	18.04 ± 3.86	13.96 ± 1.02	16.37 ± 0.61	18.66 ± 0.74	23.17 ± 3.39	<0.001

Abbreviations: SBP, systolic blood pressure; DBP, diastolic blood pressure; MAP, mean blood pressure; HR, heart rate; BMI, body mass index; WHR, waist–hip ratio; WHtR, waist–height ratio; ABSI, a body shape index; BRI, body round index; FBG, fasting blood glucose; TC, total cholesterol; TG, total triglyceride; HDL-C, high-density lipoprotein cholesterol; LDL-C, low-density lipoprotein cholesterol; BUN, blood urea nitrogen; eGFR, estimated glomerular filtration rate; AST, aspartate aminotransferase; ALT, alanine aminotransferase; DM, diabetes mellitus; baPWV, brachial-ankle pulse wave velocity.

### 3.2 Correlations of the anthropometric parameters with the CVD risk factors

A correlation analysis was conducted between obesity indices and CVD risk factors such as SBP, DBP, MAP, and levels of TC, TG, HDL-C, LDL-C, FBG, uric acid and eGFR (Supplementary Table 3)*.* MAP level positively correlated only with BMI, waist circumference, WHtR and BRI levels. ABSI positively correlated with SBP, TG and LDL-C levels, whereas negatively correlated with DBP, HDL-C and eGFR levels. BRI positively correlated with DBP, MAP, TC, TG, LDL-C, FBG, uric acid and eGFR levels, whereas negatively correlated with HDL-C levels.

### 3.3 Correlations among anthropometric indices for body composition

Correlations among anthropometric indices for body composition were highly correlated (Supplementary Table 4). Despite this, ABSI and BMI were negatively associated, suggesting ABSI was an anthropometric obesity index independent of BMI. There was an obvious positively correlation between BRI and BMI, waist circumference, WHR, WHtR, and ABSI (Supplementary Table 4).

### 3.4 Association between BMI levels and baPWV values

**Fig. 1:**
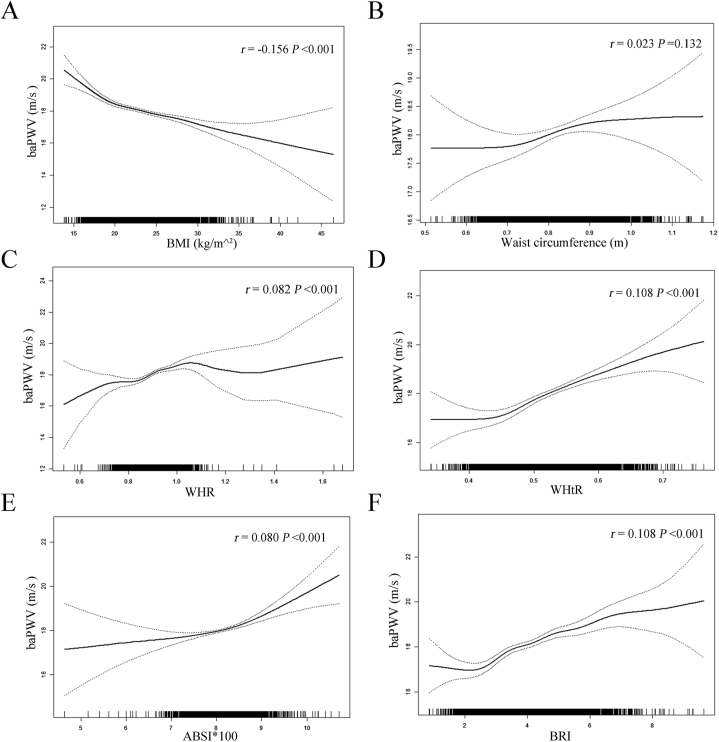
Smooth curve of correlation between different body fat parameters levels and baPWV values. Smooth curves adjusted for age, sex, MAP, HR, BMI, smoking and drinking status, DM, homocysteine, HDL-C, ALT and eGFR except the independent variable; *r* indicated coefficient of partial correlation. Abbreviations: BMI, body mass index; WHR, waist–hip ratio; WHtR, waist–height ratio; ABSI, a body shape index; BRI, body round index; baPWV, brachial-ankle pulse wave velocity.

There was an inverse correlation between BMI levels and baPWV values based on linear regression models (adjusted-*β* per SD increase: −0.42, 95% CI -0.51 to −0.33, P < 0.001; Table 2). There were lower baPWV values in the second and highest tertiles of BMI compared to BMI ≤21.70 kg/m^2^ (adjusted-*β*: −0.48, 95% CI -0.71 to −0.26, P < 0.001; adjusted-*β*: −0.98, 95% CI -1.22 to −0.74, P < 0.001, respectively; P for trend <0.001). Further confirmation of this negative association between BMI levels and baPWV was obtained by fitting smooth curves (Fig. 1A). To explore whether this inversely correlation was modified by sex, a stratified analysis as well as an interaction analysis was conducted. There was still a negative relationship between BMI levels and baPWV values across both sexes (adjusted-*β* per SD increase in male: −0.51, 95% CI -0.66 to −0.36, P < 0.001; female: −0.50, 95% CI -0.63 to −0.37, P < 0.001, respectively; Table 2).

**Table 2:** Relationship between BMI levels and baPWV values stratified by sex.

Variables	Crude Model		Model Ⅰ		Model Ⅱ	
	*β* (95%CI)	*P*-value	*β* (95%CI)	*P*-value	*β* (95%CI)	*P*-value
Total						
BMI (kg/m^^2^)						
Per *SD* increase	−0.72 (−0.88, −0.65)	<0.001	−0.37 (−0.47, −0.28)	<0.001	−0.42 (−0.51, −0.33)	<0.001
BMI tertiles						
T1 [13.83, 21.70]	*Ref*		*Ref*		*Ref*	
T2 [21.70, 24.71]	−1.02 (−1.30, −0.74)	<0.001	−0.35 (−0.57, −0.13)	0.001	−0.48 (−0.71, −0.26)	<0.001
T3 [24.71, 46.43]	−1.74 (−2.02, −1.46)	<0.001	−0.73 (−0.96, −0.50)	<0.001	−0.98 (−1.22, −0.74)	<0.001
*P-value* for trend	<0.001		<0.001		<0.001	
Male						
BMI (kg/m^^2^)						
Per *SD* increase	−0.73 (−0.89, −0.57)	<0.001	−0.37 (−0.51, −0.23)	<0.001	−0.51 (−0.66, −0.36)	<0.001
BMI tertiles						
T1 [13.83, 21.70]	*Ref*		*Ref*		*Ref*	
T2 [21.70, 24.71]	−0.89 (−1.27, −0.52)	<0.001	−0.32 (−0.62, −0.01)	0.043	−0.43 (−0.74, −0.11)	<0.001
T3 [24.71, 39.82]	−1.48 (−1.86, −1.10)	<0.001	−0.55 (−0.87, −0.23)	0.001	−0.78 (−1.13, −0.43)	<0.001
*P-value* for trend	<0.001		0.004		<0.001	
Female						
BMI (kg/m^^2^)						
Per *SD* increase	−0.83 (−0.99, −0.67)	<0.001	−0.40 (−0.52, −0.27)	<0.001	−0.50 (−0.63, −0.37)	<0.001
*P-value* for interaction	0.394		0.786		0.877	
BMI tertiles						
T1 [13.94, 21.70]	*Ref*		*Ref*		*Ref*	
T2 [21.70, 24.71]	−1.25 (−1.65, −0.84)	<0.001	−0.42 (−0.74, −0.10)	0.009	−0.55 (−0.87, −0.23)	0.004
T3 [24.71, 46.43]	−2.09 (−2.49, −1.68)	<0.001	−0.95 (−1.27, −0.63)	<0.001	−1.16 (−1.50, −0.83)	<0.001
*P-value* for trend	<0.001		<0.001		<0.001	
*P-value* for interaction	0.095		0.206		0.276	

Abbreviations: BMI, body mass index; baPWV, brachial-ankle pulse wave velocity; *Ref,* reference; *β*, effect size; CI, confidence interval; *SD*, standard deviation. ModelⅠadjusted for age, sex, MAP and HR except the subgroup variable.ModelⅡadjusted for age, sex, MAP, HR, smoking and drinking status, DM, homocysteine, HDL-C, ALT and eGFR except the subgroup variable.

### 3.5 Relationship between waist circumference levels and baPWV values

An analysis of linear regression models revealed a positive correlation between waist circumference levels and baPWVs (adjusted-*β* per SD increase: 0.16, 95% CI -0.01 to 0.33, P = 0.060; Table 3). BaPWV values were higher in the second and highest tertiles of waist circumferences relative to waist circumference ≤0.78 (adjusted-*β*: 0.38, 95% CI 0.13 to 0.63, P = 0.003; adjusted-*β*: 0.45, 95% CI 0.12 to 0.78, P = 0.008, respectively; P for trend = 0.007). Further confirmation of this positive association between waist circumference levels and baPWV was obtained by fitting smooth curves (Fig. 1B). However, the subgroup analysis found that waist circumference positively correlated with baPWV only in male hypertensive individuals in the second and highest tertiles of waist circumferences relative to waist circumference ≤0.78 (adjusted-*β*: 0.38, 95% CI 0.01 to 0.74, P = 0.043; adjusted-*β*: 0.56, 95% CI 0.07 to 1.04, P = 0.025, respectively; P for trend = 0.024, Table 3).

**Table 3:** Relationship between waist circumference levels and baPWV values stratified by sex.

Variables	Crude Model		Model Ⅰ		Model Ⅱ	
	*β* (95%CI)	*P*-value	*β* (95%CI)	*P*-value	*β* (95%CI)	*P*-value
Total						
Waist circumference (m)						
Per *SD* increase	−0.49 (−0.60, −0.38)	<0.001	−0.24 (−0.33, −0.15)	<0.001	0.16 (−0.01, 0.33)	0.060
Waist circumference tertiles						
T1 [0.51, 0.78]	*Ref*		*Ref*		*Ref*	
T2 [0.78, 0.86]	−0.40 (−0.68, −0.12)	0.005	−0.03 (−0.25, 0.19)	0.817	0.38 (0.13, 0.63)	0.003
T3 [0.87, 1.17]	−1.08 (−1.36, −0.80)	<0.001	−0.41 (−0.63, −0.19)	<0.001	0.45 (0.12, 0.78)	0.008
*P-value* for trend	<0.001		<0.001		0.007	
Male						
Waist circumference (m)						
Per *SD* increase	−0.48 (−0.63, −0.32)	<0.001	−0.27 (−0.39, −0.14)	<0.001	0.09 (−0.17, 0.34)	0.504
Waist circumference tertiles						
T1 [0.57, 0.78]	*Ref*		*Ref*		*Ref*	
T2 [0.78, 0.86]	−0.54 (−0.94, −0.14)	0.008	−0.07 (−0.39, 0.25)	0.662	0.38 (0.01, 0.74)	0.043
T3 [0.87, 0.17]	−1.05 (−1.43, −0.68)	<0.001	−0.38 (−0.69, −0.07)	0.017	0.56 (0.07, 1.04)	0.025
*P-value* for trend	<0.001		0.016		0.024	
Female						
Waist circumference (m)						
Per *SD* increase	−0.46 (−0.63, −0.29)	<0.001	−0.26 (−0.39, −0.13)	<0.001	0.17 (−0.06, 0.41)	0.149
*P-value* for interaction	0.913		0.926		0.655	
Waist circumference tertiles						
T1 [0.51, 0.78]	*Ref*		*Ref*		*Ref*	
T2 [0.78, 0.86]	−0.32 (−0.71, 0.08)	0.114	−0.03 (−0.33, 0.27)	0.842	0.33 (−0.01, 0.67)	0.061
T3 [0.87, 1.17]	−1.02 (−1.44, −0.61)	<0.001	−0.56 (−0.88, −0.24)	<0.001	0.25 (−0.22, 0.72)	0.295
*P-value* for trend	<0.001		0.001		0.024	
*P-value* for interaction	0.703		0.588		0.560	

Abbreviations: baPWV, brachial-ankle pulse wave velocity; *Ref,* reference; *β*, effect size; CI, confidence interval; *SD*, standard deviation. ModelⅠadjusted for age, sex, MAP and HR except the subgroup variable.ModelⅡadjusted for age, sex, MAP, HR, BMI, smoking and drinking status, DM, homocysteine, HDL-C, ALT and eGFR except the subgroup variable.

### 3.6 Associations between WHR, WHtR levels and baPWV values

According to linear regression models, WHR or WHtR levels were positively correlated with baPWV (adjusted-*β* per SD increase: 0.32, 95% CI 0.21 to 0.43, P < 0.001; 0.64, 95% CI 0.47 to 0.82, P < 0.001; respectively; Table 4 and Table 5). A higher baPWV value was seen in the second and highest tertile of WHR when compared to WHR ≤0.87 (adjusted-*β*: 0.53, 95% CI 0.30 to 0.76, P < 0.001; adjusted-*β*: 0.81, 95% CI 0.55 to 1.08, P < 0.001, respectively; P for trend <0.001, Table 4). Compared to WHtR ≤0.50, baPWV values were higher in the second and highest tertiles (adjusted-*β*: 0.64, 95% CI 0.39 to 0.89, P < 0.001; adjusted-*β*: 1.16, 95% CI 0.82 to 1.49, P < 0.001, respectively; P for trend <0.001, Table 4). Further confirmation of the positive correlation between WHR or WHtR levels and baPWV values was found through smooth curve fitting (Fig. 1C and D). The subgroup analysis found that the positive relationships between WHR or WHtR levels and baPWV values still existed in both sexes (Table 4, Table 5).

**Table 4:** Relationship between WHR levels and baPWV values stratified by sex.

Variables	Crude Model		Model Ⅰ		Model Ⅱ	
	*β* (95%CI)	*P*-value	*β* (95%CI)	*P*-value	*β* (95%CI)	*P*-value
Total						
WHR						
Per *SD* increase	0.09 (−0.02, 0.21)	0.115	0.04 (−0.05, 0.13)	0.408	0.32 (0.21, 0.43)	<0.001
WHR tertiles						
T1 [0.53, 0.87]	*Ref*		*Ref*		*Ref*	
T2 [0.87, 0.93]	0.01 (−0.27, 0.29)	0.928	0.14 (−0.07, 0.36)	0.197	0.53 (0.30, 0.76)	<0.001
T3 [0.93, 1.68]	0.19 (−0.09, 0.47)	0.193	0.14 (−0.08, 0.36)	0.203	0.81 (0.55, 1.08)	<0.001
*P-value* for trend	0.200		0.195		<0.001	
Male						
WHR						
Per *SD* increase	−0.11 (−0.27, 0.06)	0.205	−0.07 (−0.20, 0.07)	0.333	0.23 (0.06, 0.41)	0.008
WHR tertiles						
T1 [0.58, 0.87]	*Ref*		*Ref*		*Ref*	
T2 [0.87, 0.93]	−0.33 (−0.73, 0.06)	0.096	−0.03 (−0.34, 0.29)	0.867	0.38 (0.05, 0.72)	0.025
T3 [0.93, 1.41]	−0.20 (−0.58, 0.19)	0.311	−0.04 (−0.35, 0.26)	0.778	0.68 (0.29, 1.07)	<0.001
*P-value* for trend	0.319		0.778		<0.001	
Female						
WHR						
Per *SD* increase	0.28 (0.12, 0.44)	<0.001	0.07 (−0.05, 0.19)	0.246	0.33 (0.18, 0.47)	<0.001
*P-value* for interaction	0.001		0.134		0.417	
WHR tertiles						
T1 [0.53, 0.87]	*Ref*		*Ref*		*Ref*	
T2 [0.87, 0.93]	0.35 (−0.05, 0.75)	0.087	0.23 (−0.08, 0.53)	0.141	0.60 (0.28, 0.93)	<0.001
T3 [0.93, 1.68]	0.64 (0.24, 1.05)	0.002	0.20 (−0.12, 0.51)	0.221	0.81 (0.44, 1.17)	<0.001
*P-value* for trend	0.002		0.195		<0.001	
*P-value* for interaction	0.008		0.439		0.646	

Abbreviations: WHR, waist–hip ratio; baPWV, brachial-ankle pulse wave velocity; *Ref,* reference; *β*, effect size; CI, confidence interval; *SD*, standard deviation. ModelⅠadjusted for age, sex, MAP and HR except the subgroup variable.ModelⅡadjusted for age, sex, MAP, HR, BMI, smoking and drinking status, DM, homocysteine, HDL-C, ALT and eGFR except the subgroup variable.

**Table 5:** Relationship between WHtR levels and baPWV values stratified by sex.

Variables	Crude Model		Model Ⅰ		Model Ⅱ	
	*β* (95%CI)	*P*-value	*β* (95%CI)	*P*-value	*β* (95%CI)	*P*-value
Total						
WHtR						
Per *SD* increase	−0.08 (−0.19, 0.04)	0.194	−0.12 (−0.22, −0.03)	0.009	0.64 (0.47, 0.82)	<0.001
WHtR tertiles						
T1 [0.34, 0.50]	*Ref*		*Ref*		*Ref*	
T2 [0.50 0.56]	−0.30 (−0.58, −0.01)	0.040	0.04 (−0.18, 0.26)	0.728	0.64 (0.39, 0.89)	<0.001
T3 [0.56, 0.76]	−0.12 (−0.40, 0.16)	0.403	−0.13 (−0.35, 0.10)	0.260	1.16 (0.82, 1.49)	<0.001
*P-value* for trend	0.403		0.261		<0.001	
Male						
WHtR						
Per *SD* increase	−0.24 (−0.41, −0.07)	0.006	−0.14 (−0.27, −0.00)	0.048	0.75 (0.48, 1.03)	<0.001
WHtR tertiles						
T1 [0.34, 0.50]	*Ref*		*Ref*		*Ref*	
T2 [0.50 0.56]	−0.44 (−0.80, −0.08)	0.018	0.08 (−0.21, 0.37)	0.600	0.83 (0.48, 1.19)	<0.001
T3 [0.56, 0.75]	−0.41 (−0.82, −0.01)	0.046	−0.17 (−0.50, 0.15)	0.300	1.32 (0.81, 1.84)	<0.001
*P-value* for trend	0.025		0.385		<0.001	
Female						
WHtR						
Per *SD* increase	−0.09 (−0.25, 0.08)	0.300	−0.15 (−0.28, −0.03)	0.018	0.50 (0.28, 0.72)	<0.001
*P-value* for interaction	0.217		0.882		0.164	
WHtR tertiles						
T1 [0.35, 0.50]	*Ref*		*Ref*		*Ref*	
T2 [0.50 0.56]	−0.23 (−0.67, 0.22)	0.318	−0.12 (−0.46, 0.21)	0.474	0.35 (−0.02, 0.71)	0.061
T3 [0.56, 0.76]	−0.16 (−0.57, 0.26)	0.461	−0.22 (−0.54, 0.09)	0.167	0.88 (0.43, 1.33)	<0.001
*P-value* for trend	0.527		0.166		<0.001	
*P-value* for interaction	0.267		0.721		0.392	

Abbreviations: WHtR, waist–height ratio; baPWV, brachial-ankle pulse wave velocity; *Ref,* reference; *β*, effect size; CI, confidence interval; *SD*, standard deviation. ModelⅠadjusted for age, sex, MAP and HR except the subgroup variable.ModelⅡadjusted for age, sex, MAP, HR, BMI, smoking and drinking status, DM, homocysteine, HDL-C, ALT and eGFR except the subgroup variable.

### 3.7 Associations between ABSI, BRI levels and baPWV values

The ABSI level was positively correlated with the baPWV level in linear regression models (adjusted-*β* per SD increase for ABSI × 100: 0.27, 95% CI 0.18 to 0.36, P < 0.001; Table 6). Compared to ABSI × 100 ≤ 7.89, baPWV values were higher in the highest ABSI tertile (adjusted-*β*: 0.52, 95% CI 0.30 to 0.75, P < 0.001; P for trend <0.001; Table 6). There was a positive correlation between BRI levels and baPWV values (adjusted-*β* per SD increase: 0.64, 95% CI 0.46 to 0.81, P < 0.001; Table 7). BaPWV values in the second and highest tertile of BRI were higher than those in BRI ≤3.39 (adjusted-*β*: 0.62, 95% CI 0.37 to 0.87, P < 0.001; adjusted-*β*: 1.15, 95% CI 0.82 to 1.48, P < 0.001, respectively; P for trend <0.001; Table 7). Further confirmation of the positive relationship between ABSI or BRI and baPWV values was obtained by fitting smooth curves (Fig. 1E and F). The subgroup analysis found that the positive relationships between ABSI or BRI levels and baPWV values still existed in both sexes (Table 6, Table 7). In addition, the stratified analysis found that the relationships between anthropometric indices and baPWV values were still stable stratified by antihypertensive medication other than waist circumference (Supplementary Table 5).

**Table 6:** Relationship between ABSI levels and baPWV values stratified by sex.

Variables	Crude Model		Model Ⅰ		Model Ⅱ	
	*β* (95%CI)	*P*-value	*β* (95%CI)	*P*-value	*β* (95%CI)	*P*-value
Total						
ABSI*100						
Per *SD* increase	0.76 (0.65, 0.87)	<0.001	0.26 (0.17, 0.35)	<0.001	0.27 (0.18, 0.36)	<0.001
ABSI*100 tertiles						
T1 [4.63, 7.89]	*Ref*		*Ref*		*Ref*	
T2 [7.89, 8.27]	0.44 (0.17, 0.72)	0.002	0.00 (−0.21, 0.22)	0.968	0.10 (−0.12, 0.32)	0.380
T3 [8.27, 10.71]	1.60 (1.33, 1.88)	<0.001	0.47 (0.25, 0.70)	<0.001	0.52 (0.30, 0.75)	<0.001
*P-value* for trend	<0.001		<0.001		<0.001	
Male						
ABSI*100						
Per *SD* increase	0.60 (0.43, 0.77)	<0.001	0.21 (0.07, 0.35)	0.004	0.26 (0.12, 0.40)	<0.001
ABSI*100 tertiles						
T1 [4.63, 7.89]	*Ref*		*Ref*		*Ref*	
T2 [7.89, 8.27]	0.67 (0.30, 1.04)	<0.001	0.24 (−0.06, 0.53)	0.121	0.40 (0.10, 0.70)	0.010
T3 [8.27, 10.25]	1.33 (0.94, 1.71)	<0.001	0.45 (0.14, 0.76)	0.005	0.59 (0.27, 0.90)	<0.001
*P-value* for trend	<0.001		0.005		<0.001	
Female						
ABSI*100						
Per *SD* increase	0.84 (0.69, 0.99)	<0.001	0.26 (0.14, 0.39)	<0.001	0.23 (0.11, 0.36)	<0.001
*P-value* for interaction	0.042		0.550		0.753	
ABSI*100 tertiles						
T1 [5.16, 7.89]	*Ref*		*Ref*		*Ref*	
T2 [7.90, 8.27]	0.21 (−0.19, 0.62)	0.306	−0.27 (−0.58, 0.05)	0.102	−0.23 (−0.55, 0.08)	0.150
T3 [8.27, 10.71]	1.78 (1.39, 2.18)	<0.001	0.40 (0.09, 0.72)	0.013	0.38 (0.06, 0.70)	0.019
*P-value* for trend	<0.001		0.011		0.016	
*P-value* for interaction	0.006		0.044		0.014	

Abbreviations: ABSI, a body shape index; baPWV, brachial-ankle pulse wave velocity; *Ref,* reference; *β*, effect size; CI, confidence interval; *SD*, standard deviation. ModelⅠadjusted for age, sex, MAP and HR except the subgroup variable.ModelⅡadjusted for age, sex, MAP, HR, BMI, smoking and drinking status, DM, homocysteine, HDL-C, ALT and eGFR except the subgroup variable.

**Table 7:** Relationship between BRI levels and baPWV values stratified by sex.

Variables	Crude Model		Model Ⅰ		Model Ⅱ	
	*β* (95%CI)	*P*-value	*β* (95%CI)	*P*-value	*β* (95%CI)	*P*-value
Total						
BRI						
Per *SD* increase	−0.07 (−0.19, 0.05)	0.233	−0.13 (−0.22, −0.04)	0.007	0.64 (0.46, 0.81)	<0.001
BRI tertiles						
T1 [0.85, 3.39]	*Ref*		*Ref*		*Ref*	
T2 [3.39, 4.47]	−0.30 (−0.58, −0.01)	0.040	0.04 (−0.18, 0.26)	0.728	0.62 (0.37, 0.87)	<0.001
T3 [4.47, 9.64]	−0.12 (−0.40, 0.16)	0.403	−0.13 (−0.35, 0.10)	0.260	1.15 (0.82, 1.48)	<0.001
*P-value* for trend	0.425		0.252		<0.001	
Male						
BRI						
Per *SD* increase	−0.24 (−0.41, −0.06)	0.007	−0.14 (−0.28, −0.00)	0.046	0.79 (0.51, 1.08)	<0.001
BRI tertiles						
T1 [0.85, 3.39]	*Ref*		*Ref*		*Ref*	
T2 [3.39, 4.47]	−0.44 (−0.80, −0.08)	0.018	0.08 (−0.21, 0.37)	0.600	0.82 (0.47, 1.17)	<0.001
T3 [4.47, 9.29]	−0.41 (−0.82, −0.01)	0.046	−0.17 (−0.50, 0.15)	0.300	1.32 (0.81, 1.83)	<0.001
*P-value* for trend	0.027		0.372		<0.001	
Female						
BRI						
Per *SD* increase	−0.08 (−0.25, 0.08)	0.306	−0.15 (−0.28, −0.03)	0.014	0.48 (0.26, 0.70)	<0.001
*P-value* for interaction	0.204		0.910		0.091	
BRI						
T1 [0.97, 3.39]	*Ref*		*Ref*		*Ref*	
T2 [3.39, 4.47]	−0.23 (−0.67, 0.22)	0.318	−0.12 (−0.46, 0.21)	0.474	0.33 (−0.03, 0.69)	0.071
T3 [4.47, 9.64]	−0.16 (−0.57, 0.26)	0.461	−0.22 (−0.54, 0.09)	0.167	0.88 (0.43, 1.32)	<0.001
*P-value* for trend	0.537		0.166		<0.001	
*P-value* for interaction	0.649		0.650		0.160	

Abbreviations: BRI, body round index; baPWV, brachial-ankle pulse wave velocity; *Ref,* reference; *β*, effect size; CI, confidence interval; *SD*, standard deviation. ModelⅠadjusted for age, sex, MAP and HR except the subgroup variable.ModelⅡadjusted for age, sex, MAP, HR, BMI, smoking and drinking status, DM, homocysteine, HDL-C, ALT and eGFR except the subgroup variable.

### 3.8 Relationship between anthropometric indices and arterial stiffness

The relationship between anthropometric indices and arterial stiffness based on baPWV values were also consistent as before (Supplementary Table 6). To determine the most parsimonious model for prediction of arterial stiffness based on baPWV values, we plotted receiver operating characteristic curves for the six anthropometric indices. Compared to other indices, ABSI had the highest predictive power of arterial stiffness (area under the curve, 0.594; P < 0.001, Supplementary Figure 1).

## 4 Discussion

Subclinical atherosclerosis is associated with effective stratification of CVD risks based on arterial stiffness in both non-hypertensive and hypertensive population [28,50]. As a result, determining arterial stiffness factors is crucial for preventing and treating cardiovascular disease. Attention increases to body fat parameters related to obesity in consideration of that the established association between obesity and CVD [3,29,51]. Health care workers should consistently reduce the risk of CV events across high-risk groups such as hypertensive population by preventing and controlling obesity. The identification of a simple and effective method of identifying visceral obesity is therefore very important.

Our study examined the relationship between obesity indices and arterial stiffness as a risk factor for CV events. In this cross-sectional study, six anthropometric indices were examined in relation to arterial stiffness in Chinese adults with hypertension. Chinese adults with hypertension were found to have inverse associations between BMI and baPWV, while positive associations were found for WHR, WHtR, ABSI and BRI. Furthermore, waist circumference positively correlated with baPWV only in male hypertensive individuals. Also, visceral adiposity predominates in men, while subcutaneous fat predominates in women [52]. The sex-specific disparities between waist circumference and arterial stiffness could be interpreted by that waist circumference cannot distinguish between visceral and subcutaneous fats [48]. Compared to other indices, ABSI had the highest predictive power of arterial stiffness.

These finding suggested that the visceral obesity indices are strongly associated with arterial stiffness than BMI.

The relationship of BMI with PWV is still debatable among hypertensive patients [[34], [35], [36], [37], [38], [39], [40]]. Studies have reported insignificant correlation [34,38], positive correlation [35,37,39], or negative correlation [36,40]. These apparent discrepancies can be explained by a number of factors, including sample size, ethnicity, and region. In addition, BMI does not differentiate between fat mass and fat-free muscle, reflect visceral and subcutaneous fat distributions, or account for sex differences in fat content, which may partially explain our results [3,4,6].

Waist circumference, WHR and WHtR are regional anthropometric indices of abdominal obesity that are less influenced by muscle and bone mass, and might be better at predicting CV events and mortality than BMI [3,7,[53], [54], [55]]. Obesity measured by abdominal circumference, but not BMI, was associated with an increased risk of cardiovascular death [42]. Visceral adiposity was positively associated with endothelial dysfunction and vascular stiffness [56,57]. There is a strong association between waist circumference, WHR, and WHtR in hypertensive patients but there is a weak relationship between BMI and cfPWV [39]. Whitehall II study found that central adiposity by waist circumference and WHR is a significant and potentially modifiable determinant of vascular stiffness [30,58]. Using bioelectrical impedance analysis, Kim et al. [29] found that baPWV was correlated with WHR and visceral fat area (VFA), but not with BMI and waist circumference measurements. In an analysis of 146 middle-aged participants, cfPWV and baPWV had stronger relationships with waist circumference and VFA than with BMI [59]. Our findings, along with previous studies, suggested that there is a stronger relationship between abdominal adiposity and arterial stiffness than overall obesity.

It appears that ABSI reflects visceral adiposity independent of BMI and is a significant marker of arterial stiffening in diabetics [60]. It was found that ABSI had a stronger association with total mortality, cardiovascular mortality, and cancer mortality than BMI, waist circumference, and WHtR [60]. According to a large European cohort study, ABSI achieves better mortality risk stratification with linear associations than alternative abdominal obesity indices [61]. ABSI, however, was found to be a weaker index for identifying CVD risk than BMI and waist circumference [10,62]. These discrepancies could be explained by various sizes and characteristics of the study sample, racial differences.

The baPWV has been associated with ABSI in both sexes in Korea [9]. Zhang et al. [48] found that ABSI positively correlates to baPWV, but the area under curve value for ABSI was lower than that for WHtR in recognizing arterial stiffness in the Chinese population. ABSI was weakly correlated with cfPWV in a study consisting of 1442 obese and overweight Chinese adults, but not significantly more than classical obesity parameters [47]. When compared to other body fat parameters, ABSI had the highest discriminatory power in predicting high cardio-ankle vascular index (CAVI), while BMI was inversely related to baPWV [12]. CAVI and metabolic syndrome were positively associated with ABSI in the middle-aged population, and it helped to discriminate subjects with arterial stiffness [63,64]. In a study of 7127 asymptomatic Korean participants, increased ABSI rather than waist circumference was positively associated with high CAVI [65]. These results suggested that among classical obesity indices, ABSI has an advantage because of its being independent of BMI. ABSI had the highest predictive power of arterial stiffness based on baPWV in our analysis (Supplemenary Fig. 1). Thus, ABSI is an easily calculated index of visceral adiposity and may be effective in primary health care even without medical equipment measuring visceral adiposity.

The BRI is considered as a better metric of visceral adiposity than BMI and waist circumference [66]. BRI is still disputed as a predictor of CVD risk, however [48]. A few studies have indicated that BRI can be used to predict diabetes and CV health [15,67]. While other studies suggested that BRI was not superior to classic obesity indices for identifying CVD [46,68]. PERSIAN cohort study indicated that waist circumference, WHR, WHtR and BRI were positively associated with cfPWV in healthy middle-aged adults [69]. Choi et al. [9] found that only women in the Korean population had a positive correlation between BRI and baPWV. Zhang et al. [48] found that there was a significant relationship between WHtR, ABSI, and BRI and between WHtR and BRI and arterial stiffness in both sexes, respectively. Li et al. [47] found that BRI have the highest association with cfPWV compared to other anthropometric indices in overweight and obese adults. According to another cross-sectional study among rural Chinese, BRI, not ABSI, was a superior indicator to discriminate left ventricular hypertrophy from other classical obesity indicators [10]. BRI may be a better indicator of arterial stiffness than other anthropometric indices, possibly due to its strong correlation with visceral fat area [47].

The aim of ABSI or BRI was to develop as traditional obesity indices fail to precisely reflect abdominal adipose fat [47]. Similar to previous studies [47], we found ABSI is completely independent of BMI, and BRI has a certain correlation with BMI. Overall, the above results suggest that ABSI and BRI represent the excess risk from high visceral adiposity that is complementary to classical obesity indices. Thus, ABSI and BRI could be used as novel useful indices of visceral obesity for the diagnosis of metabolic syndrome and assessment of CVD risk due to its simplicity and rich clinical practice.

Ji et al. [70] also found that baPWV was correlated with WHR and visceral fat area, but not with BMI and waist circumference in eastern Asian populations. Remnant cholesterol was independently associated with joint arteriosclerosis and atherosclerosis progression measured by baPWV beyond LDL-C [71]. As body fat is closely related with lipids, we hold opinion that lipids had the potential mediating effects at the relationship between waist-related indices and baPWV.

The study has some limitations. First, our study focuses only on the Chinese hypertensive adults and therefore, it remains to be determined whether the results can be generalized to other ethnic groups or regions. Second, the clinical characteristics were gathered by questionnaires and thus, the quality of information disclosure was deeply influenced by the attitude and educational level of hypertensive population. In addition, due to the cross-sectional nature of the study, we cannot establish a cause-effect relationship between obesity indices and baPWV. It has been established that weight loss reduces arterial stiffness, and its magnitude is correlated with the loss of total and abdominal adiposity [72]. Further researches are needed to determine whether weight loss improves arterial stiffness and reduces the risk of CV events.

In conclusion, the results of this cross-sectional study showed that BMI was negatively associated with mean baPWV, while WHR, WHtR, ABSI, and BRI were positively associated in both sexes in Chinese adults with hypertension. Furthermore, waist circumference positively correlated with mean baPWV only in male hypertensive individuals. It has implications to employ abdominal obesity instead of overall obesity related indices for the diagnosis of metabolic syndrome and assessment of CVD risk in Chinese adults with hypertension.

## Financial support

This work was supported by the Cultivation of backup projects for National Science and Technology Awards (20223AEI91007), Jiangxi Science and Technology Innovation Base Plan - Jiangxi Clinical Medical Research Center (20223BCG74012), Science and Technology Innovation Base Construction Project (20221ZDG02010), Jiangxi Provincial Natural Science Foundation (20212ACB206019, 20224BAB206090, 20232BAB206140，20232ACB216006), 10.13039/501100010847Jiangxi Provincial Health Commission Science and Technology Project (202130440,202210495,202310528), Jiangxi Provincial Drug Administration Science and Technology Project (2022JS41，2023JS26), Fund project of the Second Affiliated Hospital of Nanchang University（2016YNQN12034，2019YNLZ12010，2021efyA01，2021YNFY2024）.

## CRediT authorship contribution statement

**Feng Hu:** Writing – original draft, Software, Methodology, Investigation, Conceptualization. **Wei Zhou:** Visualization, Validation, Supervision, Resources, Project administration, Methodology, Formal analysis. **Tao Wang:** Visualization, Validation, Project administration, Investigation, Data curation. **Chao Yu:** Visualization, Software, Project administration, Methodology. **Lingjuan Zhu:** Validation, Supervision, Resources, Project administration, Formal analysis, Data curation. **Huihui Bao:** Writing – original draft, Validation, Software, Resources, Investigation, Funding acquisition. **Xiaoshu Cheng:** Writing – review & editing, Resources, Project administration, Methodology, Funding acquisition, Data curation, Conceptualization.

## Declaration of competing interest

The authors declare that they have no known competing financial interests or personal relationships that could have appeared to influence the work reported in this paper.
