# Supplementary material for: Association between six different types of anthropometric indices and arterial stiffness measured by brachial-ankle pulse wave velocity in hypertensive Chinese adults
Source: Heliyon. 2024 Mar 27;10(7):e28523. doi: 10.1016/j.heliyon.2024.e28523 (PMC11004534; doi:10.1016/j.heliyon.2024.e28523)
Supplement: Multimedia component 1 [file mmc1.docx]

**Supplemenary Table Legends**

| **Supplementary Table 1. Association of covariates with the baPWV levels.** | | |
| --- | --- | --- |
| **Covariates** | **baPWV** | |
|  | ***β* (95%CI)** | ***P-value*** |
| Age (years) | 0.18 (0.17, 0.19) | <0.001 |
| Sex |  |  |
| Male | *Ref* | |
| Female |  | <0.001 |
| SBP (mmHg) | 0.10 (0.09, 0.10) | <0.001 |
| DBP (mmHg) | 0.05 (0.04, 0.06) | <0.001 |
| MAP (mmHg) | 0.10 (0.09, 0.11) | <0.001 |
| HR (times/min) | 0.06 (0.05, 0.07) | <0.001 |
| BMI (kg/m^2) | -0.22 (-0.25, -0.19) | <0.001 |
| Waist circumference (m) | -5.10 (-6.29, -3.92) | <0.001 |
| WHtR | -1.24 (-3.11, 0.63) | 0.194 |
| WHR | 1.29 (-0.31, 2.90) | 0.115 |
| ABSI*100 | 1.57 (1.34, 1.80) | <0.001 |
| BRI | -0.06 (-0.15, 0.04) | 0.233 |
| Smoking status, n(%) |  |  |
| Never | *Ref* | |
| Former smoker | 0.00 (-0.32, 0.33) | 0.978 |
| Current smoker | -0.47 (-0.74, -0.21) | <0.001 |
| Drinking status, n(%) |  |  |
| Never | *Ref* | |
| Former drinker | -0.06 (-0.45, 0.32) | 0.746 |
| Current drinker | -0.49 (-0.76, -0.22) | <0.001 |
| Homocysteine (μmol/L) | 0.02 (0.01, 0.03) | <0.001 |
| TC (mmol/L) | 0.17 (0.07, 0.27) | 0.001 |
| TG (mmol/L) | -0.02 (-0.10, 0.07) | 0.717 |
| HDL-C (mmol/L) | 0.64 (0.35, 0.93) | <0.001 |
| LDL-C (mmol/L) | -0.00 (-0.15, 0.14) | 0.984 |
| eGFR (ml/min/1.73m^2) | -0.04 (-0.05, -0.04) | <0.001 |
| AST (U/L) | 0.01 (-0.00, 0.01) | 0.243 |
| ALT (U/L) | -0.02 (-0.03, -0.01) | <0.001 |
| DM, n(%) |  |  |
| No | *Ref* | |
| Yes | 0.72 (0.42, 1.02) | <0.001 |
| Antihypertensive medications, n(%) |  |  |
| No | *Ref* | |
| Yes | -0.01 (-0.25, 0.22) | 0.906 |
| Lipid-lowering agents, n(%) |  |  |
| No | *Ref* | |
| Yes | -0.91 (-1.77, -0.04) | 0.040 |
| Antiplatelet agents, n(%) |  |  |
| No | *Ref* | |
| Yes | 0.65 (-0.25, 1.55) | 0.158 |
| Abbreviations: SBP, systolic blood pressure; DBP, diastolic blood pressure; MAP, mean blood pressure; HR, heart rate; BMI, body mass index; ABSI, a body shape index; VAI, visceral adiposity index; BRI, body round index; TC, total cholesterol; TG, total triglyceride; HDL-C, high-density lipoprotein cholesterol; LDL-C, low-density lipoprotein cholesterol; BUN, blood urea nitrogen; eGFR, estimated glomerular filtration rate; AST, aspartate aminotransferase; ALT, alanine aminotransferase; DM, diabetes mellitus; baPWV, brachial-ankle pulse wave velocity. Values are regression coefficients from univariate regression models and reflect differences in the outcomes of interest per unit change of each covariate and for different categories of each covariate as compared to the reference group. | | |

| **Supplementary Table 2. Clinical characteristics of participants grouped by sex.** | | | |
| --- | --- | --- | --- |
| **Characteristics** | **Male** | **Female** | ***P-value*** |
| Number of subjects (n) | 2131 | 2191 |  |
| Age (years) | 64.23 ± 9.91 | 64.07 ± 9.17 | 0.575 |
| SBP (mmHg) | 145.54 ± 17.83 | 148.92 ± 16.99 | <0.001 |
| DBP (mmHg) | 90.22 ± 11.30 | 87.82 ± 10.29 | <0.001 |
| MAP (mmHg) | 108.66 ± 11.85 | 108.18 ± 10.89 | 0.168 |
| HR (times/min) | 73.96 ± 14.27 | 77.61 ± 14.28 | <0.001 |
| Height (m) | 1.61 ± 0.06 | 1.50 ± 0.06 | <0.001 |
| Weight (Kg) | 60.52 ± 10.68 | 53.43 ± 9.53 | <0.001 |
| BMI (kg/m^^2^) | 23.15 ± 3.40 | 23.56 ± 3.62 | <0.001 |
| Waist circumference (m) | 0.83 ± 0.10 | 0.82 ± 0.09 | <0.001 |
| Hipline (m) | 0.92 ± 0.07 | 0.91 ± 0.07 | <0.001 |
| WHtR | 0.51 ± 0.06 | 0.54 ± 0.06 | <0.001 |
| WHR | 0.90 ± 0.07 | 0.89 ± 0.07 | <0.001 |
| ABSI*100 | 8.06 ± 0.44 | 8.12 ± 0.52 | <0.001 |
| BRI | 3.69 ± 1.11 | 4.27 ± 1.27 | <0.001 |
| Smoking status, n(%) |  |  | <0.001 |
| Never | 416 (19.52%) | 1913 (87.31%) |  |
| Former smoker | 607 (28.48%) | 117 (5.34%) |  |
| Current smoker | 1108 (51.99%) | 161 (7.35%) |  |
| Drinking status, n(%) |  |  | <0.001 |
| Never | 823 (38.62%) | 1923 (87.77%) |  |
| Former drinker | 345 (16.19%) | 102 (4.66%) |  |
| Current drinker | 963 (45.19%) | 166 (7.58%) |  |
| Homocysteine (μmol/L) | 16.51 (13.52-21.97) | 13.75 (11.85-17.07) | <0.001 |
| FBG (mmol/L) | 6.05 ± 1.59 | 6.21 ± 1.66 | <0.001 |
| TC (mmol/L) | 4.96 ± 1.06 | 5.34 ± 1.11 | <0.001 |
| TG (mmol/L) | 1.65 ± 1.32 | 1.91 ± 1.30 | <0.001 |
| HDL-C (mmol/L) | 1.47 ± 0.41 | 1.52 ± 0.39 | <0.001 |
| LDL-C (mmol/L) | 2.82 ± 0.76 | 3.07 ± 0.80 | <0.001 |
| Uric acid (mmol/L) | 475.60 ± 120.42 | 386.67 ± 105.13 | <0.001 |
| Creatinine (mmol/L) | 77.00 (67.00-93.00) | 57.00 (50.00-68.00) | <0.001 |
| BUN (mmol/L) | 5.55 ± 1.96 | 5.27 ± 1.60 | <0.001 |
| eGFR (ml/min/1.73m^2) | 84.67 ± 20.21 | 89.02 ± 18.35 | <0.001 |
| Total bilirubin (mmol/L) | 15.30 ± 6.79 | 13.49 ± 5.87 | <0.001 |
| Direct bilirubin (mmol/L) | 5.79 ± 2.18 | 4.93 ± 1.80 | <0.001 |
| AST (U/L) | 25.00 (21.00-31.00) | 24.00 (20.00-29.00) | <0.001 |
| ALT (U/L) | 18.00 (13.00-26.00) | 16.00 (12.00-22.00) | <0.001 |
| DM, n(%) | 335 (15.72%) | 433 (19.76%) | <0.001 |
| Dyslipidemia, n(%) | 670 (31.44%) | 897 (40.94%) | <0.001 |
| Antihypertensive drugs, n(%) | 1272 (59.69%) | 1263 (57.64%) | 0.172 |
| Hypoglycemic agents, n(%) | 74 (3.47%) | 96 (4.38%) | 0.124 |
| Lipid-lowering agents, n(%) | 28 (1.31%) | 50 (2.28%) | 0.017 |
| Antiplatelet agents, n(%) | 35 (1.64%) | 37 (1.69%) | 0.905 |
| Ankle brachial index | 1.16 ± 0.08 | 1.14 ± 0.07 | <0.001 |
| baPWV (m/s) | 17.73 ± 3.70 | 18.34 ± 3.98 | <0.001 |
| Abbreviations: SBP, systolic blood pressure; DBP, diastolic blood pressure; MAP, mean blood pressure; HR, heart rate; BMI, body mass index; WHR, waist–hip ratio; WHtR, waist–height ratio; ABSI, a body shape index; BRI, body round index; FBG, fasting blood glucose; TC, total cholesterol; TG, total triglyceride; HDL-C, high-density lipoprotein cholesterol; LDL-C, low-density lipoprotein cholesterol; BUN, blood urea nitrogen; eGFR, estimated glomerular filtration rate; AST, aspartate aminotransferase; ALT, alanine aminotransferase; DM, diabetes mellitus; baPWV, brachial-ankle pulse wave velocity. | | | |

| **Supplementary Table 3. Correlations of the anthropometric parameters with the CVD risk factors.** | | | | | | | | | | |
| --- | --- | --- | --- | --- | --- | --- | --- | --- | --- | --- |
| Characteristics | SBP | DBP | MAP | TC | TG | HDL-C | LDL-C | FBG | Uric acid | eGFR |
| BMI | -0.041** | 0.195*** | 0.103*** | 0.107*** | 0.288*** | -0.255*** | 0.236*** | 0.161*** | 0.147*** | 0.115*** |
| WC | -0.033* | 0.178*** | 0.096*** | 0.064*** | 0.288*** | -0.320*** | 0.216*** | 0.162*** | 0.198*** | 0.074*** |
| WHR | -0.007 | 0.063*** | 0.037* | 0.068*** | 0.220*** | -0.239*** | 0.172*** | 0.161*** | 0.185*** | -0.020 |
| WHtR | 0.032* | 0.095*** | 0.077*** | 0.113*** | 0.278*** | -0.262*** | 0.240*** | 0.152*** | 0.082*** | 0.062*** |
| ABSI | 0.070*** | -0.060*** | -0.002 | -0.008 | 0.069*** | -0.143*** | 0.050** | 0.0331* | 0.026 | -0.058*** |
| BRI | 0.031* | 0.088*** | 0.072*** | 0.113*** | 0.272*** | -0.251*** | 0.235*** | 0.151*** | 0.081*** | 0.059*** |
| Notes: *p <0.05, **p <0.01, ***p <0.001, for the correlation coefficient. Abbreviations: SBP, systolic blood pressure; DBP, diastolic blood pressure;MAP, mean blood pressure; TC, total cholesterol; TG, total triglyceride; HDL-C, high-density lipoprotein cholesterol; LDL-C, low-density lipoprotein cholesterol; FBG, fasting blood glucose; eGFR, estimated glomerular filtration rate; BMI, body mass index; WC, waist circumference; WHR, waist–hip ratio; WHtR, waist–height ratio; ABSI, a body shape index; BRI, body round index. | | | | | | | | | | |

| **Supplementary Table 4. Correlations among anthropometric indices for body composition.** | | | | | | |
| --- | --- | --- | --- | --- | --- | --- |
| Characteristics | BMI | WC | WHR | WHtR | ABSI | BRI |
| BMI | 1 | 0.836*** | 0.547*** | 0.817*** | -0.045** | 0.818*** |
| WC | 0.836*** | 1 | 0.770*** | 0.895*** | 0.452*** | 0.888*** |
| WHR | 0.547*** | 0.770*** | 1 | 0.758*** | 0.584*** | 0.745*** |
| WHtR | 0.817*** | 0.895*** | 0.758*** | 1 | 0.488*** | 0.997*** |
| ABSI | -0.045** | 0.452*** | 0.584*** | 0.488*** | 1 | 0.477*** |
| BRI | 0.818*** | 0.888*** | 0.745*** | 0.997*** | 0.477*** | 1 |
| Notes: *p <0.05, **p <0.01, ***p <0.001, for the correlation coefficient. Abbreviations: BMI, body mass index; WC, waist circumference; WHR, waist–hip ratio; WHtR, waist–height ratio; ABSI, a body shape index; BRI, body round index. | | | | | | |

| **Supplementary Table 5. Relationship between anthropometric indices and baPWV values stratified by antihypertensive medication.** | | | | | | | | |
| --- | --- | --- | --- | --- | --- | --- | --- | --- |
| **Variables** | **Crude Model** | |  | **Model Ⅰ** | |  | **Model Ⅱ** | |
|  | ***β* (95%CI)** | ***P-value*** |  | ***β* (95%CI)** | ***P-value*** |  | ***β* (95%CI)** | ***P-value*** |
| Without antihypertensive drugs |  |  |  |  |  |  |  |  |
| BMI (kg/m^^2^) |  |  |  |  |  |  |  |  |
| Per *SD* increase | -0.89 (-1.06, -0.73) | <0.001 |  | -0.39 (-0.54, -0.25) | <0.001 |  | -0.52 (-0.67, -0.36) | <0.001 |
| BMI tertiles |  |  |  |  |  |  |  |  |
| T1 [13.83, 21.70] | *Ref* | |  | *Ref* | |  | *Ref* | |
| T2 [21.70, 24.71] | -1.04 (-1.46, -0.63) | <0.001 |  | -0.36 (-0.70, -0.03) | 0.035 |  | -0.48 (-0.82, -0.14) | 0.006 |
| T3 [24.71, 46.43] | -1.99 (-2.40, -1.57) | <0.001 |  | -0.76 (-1.12, -0.40) | <0.001 |  | -0.99 (-1.36, -0.61) | <0.001 |
| Waist circumference (m) |  |  |  |  |  |  |  |  |
| Per *SD* increase | -0.54 (-0.72, -0.37) | <0.001 |  | -0.18 (-0.33, -0.04) | 0.012 |  | 0.37 (0.11, 0.63) | 0.006 |
| Waist circumference tertiles |  |  |  |  |  |  |  |  |
| T1 [0.52, 0.78] | *Ref* | |  | *Ref* | |  | *Ref* | |
| T2 [0.78, 0.86] | -0.47 (-0.88, -0.05) | 0.028 |  | -0.00 (-0.33, 0.33) | 0.985 |  | 0.46 (0.09, 0.84) | 0.016 |
| T3 [0.87, 1.17] | -1.29 (-1.72, -0.86) | <0.001 |  | -0.37 (-0.72, -0.02) | 0.040 |  | 0.62 (0.10, 1.13) | 0.019 |
| WHR |  |  |  |  |  |  |  |  |
| Per *SD* increase | 0.17 (-0.02, 0.35) | 0.074 |  | 0.10 (-0.04, 0.25) | 0.163 |  | 0.44 (0.26, 0.62) | <0.001 |
| WHR tertiles |  |  |  |  |  |  |  |  |
| T1 [0.67, 0.87] | *Ref* | |  | *Ref* | |  | *Ref* | |
| T2 [0.87, 0.93] | 0.23 (-0.18, 0.65) | 0.271 |  | 0.34 (0.02, 0.67) | 0.039 |  | 0.78 (0.44, 1.13) | <0.001 |
| T3 [0.93, 1.35] | 0.41 (-0.03, 0.85) | 0.068 |  | 0.32 (-0.02, 0.67) | 0.067 |  | 1.11 (0.69, 1.52) | <0.001 |
| WHtR |  |  |  |  |  |  |  |  |
| Per *SD* increase | -0.09 (-0.27, 0.09) | 0.308 |  | -0.04 (-0.18, 0.11) | 0.629 |  | 0.89 (0.64, 1.15) | <0.001 |
| WHtR tertiles |  |  |  |  |  |  |  |  |
| T1 [0.34, 0.50] | *Ref* | |  | *Ref* | |  | *Ref* | |
| T2 [0.50 0.56] | -0.29 (-0.72, 0.13) | 0.174 |  | 0.14 (-0.19, 0.48) | 0.402 |  | 0.81 (0.44, 1.18) | <0.001 |
| T3 [0.56, 0.76] | -0.13 (-0.56, 0.30) | 0.552 |  | -0.01 (-0.35, 0.34) | 0.964 |  | 1.41 (0.91, 1.92) | <0.001 |
| ABSI*100 |  |  |  |  |  |  |  |  |
| Per *SD* increase | 0.96 (0.79, 1.13) | <0.001 |  | 0.43 (0.29, 0.57) | <0.001 |  | 0.42 (0.28, 0.56) | <0.001 |
| ABSI*100 tertiles |  |  |  |  |  |  |  |  |
| T1 [4.63, 7.89] | *Ref* | |  | *Ref* | |  | *Ref* | |
| T2 [7.89, 8.27] | 0.83 (0.41, 1.25) | <0.001 |  | 0.21 (-0.12, 0.55) | 0.211 |  | 0.26 (-0.07, 0.60) | 0.128 |
| T3 [8.27, 10.25] | 1.98 (1.56, 2.40) | <0.001 |  | 0.77 (0.43, 1.12) | <0.001 |  | 0.77 (0.43, 1.12) | <0.001 |
| BRI |  |  |  |  |  |  |  |  |
| Per *SD* increase | -0.09 (-0.27, 0.09) | 0.343 |  | -0.04 (-0.18, 0.11) | 0.600 |  | 0.91 (0.65, 1.17) | <0.001 |
| BRI tertiles |  |  |  |  |  |  |  |  |
| T1 [0.85, 3.39] | *Ref* | |  | *Ref* | |  | *Ref* | |
| T2 [3.40, 4.47] | -0.29 (-0.72, 0.13） | 0.174 |  | 0.14 (-0.19, 0.48) | 0.402 |  | 0.81 (0.44, 1.18) | <0.001 |
| T3 [4.47, 9.64] | -0.13 (-0.56, 0.30) | 0.552 |  | -0.01 (-0.35, 0.34) | 0.964 |  | 1.41 (0.91, 1.92) | <0.001 |
| With antihypertensive drugs |  |  |  |  |  |  |  |  |
| BMI (kg/m^^2^) |  |  |  |  |  |  |  |  |
| Per *SD* increase | -0.67 (-0.83, -0.52) | <0.001 |  | -0.40 (-0.53, -0.28) | <0.001 |  | -0.52 (-0.65, -0.39) | <0.001 |
| BMI tertiles |  |  |  |  |  |  |  |  |
| T1 [13.94, 21.70] | *Ref* | |  | *Ref* | |  | *Ref* | |
| T2 [21.70, 24.71] | -1.00 (-1.37, -0.62) | <0.001 |  | -0.43 (-0.72, -0.13) | 0.005 |  | -0.54 (-0.84, -0.24) | 0.005 |
| T3 [24.72, 40.84] | -1.59 (-1.96, -1.21) | <0.001 |  | -0.82 (-1.12, -0.52) | <0.001 |  | -1.04 (-1.36, -0.71) | <0.001 |
| Waist circumference (m) |  |  |  |  |  |  |  |  |
| Per *SD* increase | -0.46 (-0.61, -0.31) | <0.001 |  | -0.32 (-0.44, -0.20) | <0.001 |  | 0.00 (-0.22, 0.23) | 0.969 |
| Waist circumference tertiles |  |  |  |  |  |  |  |  |
| T1 [0.51, 0.78] | *Ref* | |  | *Ref* | |  | *Ref* | |
| T2 [0.78, 0.86] | -0.35 (-0.73, 0.03) | 0.070 |  | -0.09 (-0.39, 0.20) | 0.530 |  | 0.29 (-0.04, 0.63) | 0.084 |
| T3 [0.87, 1.17] | -0.94 (-1.31, -0.57) | <0.001 |  | -0.52 (-0.81, -0.23) | <0.001 |  | 0.31 (-0.13, 0.75) | 0.166 |
| WHR |  |  |  |  |  |  |  |  |
| Per *SD* increase | 0.05 (-0.10, 0.20) | 0.528 |  | -0.02 (-0.13, 0.10) | 0.740 |  | 0.25 (0.11, 0.39) | <0.001 |
| WHR tertiles |  |  |  |  |  |  |  |  |
| T1 [0.53, 0.87] | *Ref* | |  | *Ref* | |  | *Ref* | |
| T2 [0.87, 0.93] | -0.16 (-0.54, 0.22) | 0.402 |  | -0.02 (-0.32, 0.27) | 0.884 |  | 0.36 (0.05, 0.67) | <0.001 |
| T3 [0.93, 1.68] | 0.03 (-0.34, 0.40) | 0.872 |  | -0.03 (-0.31, 0.26) | 0.859 |  | 0.62 (0.27, 0.96) | <0.001 |
| WHtR |  |  |  |  |  |  |  |  |
| Per *SD* increase | -0.06 (-0.22, 0.09) | 0.402 |  | -0.21 (-0.33, -0.09) | <0.001 |  | 0.44 (0.21, 0.67) | <0.001 |
| WHtR tertiles |  |  |  |  |  |  |  |  |
| T1 [0.34, 0.50] | *Ref* | |  | *Ref* | |  | *Ref* | |
| T2 [0.50 0.56] | -0.30 (-0.67, 0.08) | 0.127 |  | -0.10 (-0.39, 0.19) | 0.503 |  | 0.46 (0.12, 0.79) | 0.007 |
| T3 [0.56, 0.75] | -0.11 (-0.49, 0.26) | 0.555 |  | -0.29 (-0.59, 0.01) | 0.056 |  | 0.93 (0.49, 1.37) | <0.001 |
| ABSI*100 |  |  |  |  |  |  |  |  |
| Per *SD* increase | 0.62 (0.46, 0.77) | <0.001 |  | 0.14 (0.02, 0.26) | 0.027 |  | 0.15 (0.02, 0.27) | 0.020 |
| ABSI*100 tertiles |  |  |  |  |  |  |  |  |
| T1 [5.16, 7.89] | *Ref* | |  | *Ref* | |  | *Ref* | |
| T2 [7.90, 8.27] | 0.16 (-0.21, 0.53) | 0.400 |  | -0.16 (-0.45, 0.13) | 0.285 |  | -0.07 (-0.36, 0.22) | 0.636 |
| T3 [8.27, 10.71] | 1.33 (0.96, 1.70) | <0.001 |  | 0.25 (-0.04, 0.54) | 0.097 |  | 0.30 (0.00, 0.59) | 0.050 |
| BRI |  |  |  |  |  |  |  |  |
| Per *SD* increase | -0.06 (-0.21, 0.09) | 0.446 |  | -0.22 (-0.34, -0.10) | <0.001 |  | 0.43 (0.20, 0.66) | <0.001 |
| BRI tertiles |  |  |  |  |  |  |  |  |
| T1 [0.85, 3.39] | *Ref* | |  | *Ref* | |  | *Ref* | |
| T2 [3.39, 4.47] | -0.30 (-0.67, 0.08) | 0.127 |  | -0.10 (-0.39, 0.19) | 0.503 |  | 0.46 (0.12, 0.79) | 0.007 |
| T3 [4.47, 9.28] | -0.11 (-0.49, 0.26) | 0.555 |  | -0.29 (-0.59, 0.01) | 0.056 |  | 0.93 (0.49, 1.37) | <0.001 |
| Abbreviations:BMI, body mass index; WC, waist circumference; WHR, waist–hip ratio; WHtR, waist–height ratio; ABSI, a body shape index; BRI, body round index; baPWV, brachial-ankle pulse wave velocity; *Ref,* reference; *β*, effect size; CI, confidence interval; *SD*, standard deviation. ModelⅠadjusted for age, sex, MAP and HR except the subgroup variable. ModelⅡadjusted for age, sex, MAP, HR, BMI, smoking and drinking status, DM, homocysteine, HDL-C, ALT and eGFR except the subgroup variable. | | | | | | | | |

| **Supplementary Table 6. Relationship between anthropometric indices and arterial stiffness based on baPWV values.** | | | | | | | | |
| --- | --- | --- | --- | --- | --- | --- | --- | --- |
| **Variables** | **Crude Model** | |  | **Model Ⅰ** | |  | **Model Ⅱ** | |
|  | ***OR (95%CI)*** | ***P-value*** |  | ***OR (95%CI)*** | ***P-value*** |  | ***OR (95%CI)*** | ***P-value*** |
| BMI (kg/m^^2^) |  |  |  |  |  |  |  |  |
| Per *SD* increase | 0.75 (0.70, 0.80) | <0.001 |  | 0.89 (0.82, 0.96) | 0.002 |  | 0.80 (0.73, 0.87) | <0.001 |
| BMI tertiles |  |  |  |  |  |  |  |  |
| T1 [13.83, 21.70] | *Ref* | |  | *Ref* | |  | *Ref* | |
| T2 [21.70, 24.71] | 0.68 (0.59, 0.79) | <0.001 |  | 0.90 (0.75, 1.07) | 0.224 |  | 0.79 (0.66, 0.95) | 0.011 |
| T3 [24.71, 46.43] | 0.51 (0.44, 0.59) | <0.001 |  | 0.77 (0.64, 0.93) | 0.007 |  | 0.62 (0.51, 0.76) | <0.001 |
| Waist circumference (m) |  |  |  |  |  |  |  |  |
| Per *SD* increase | 0.85 (0.80, 0.91) | <0.001 |  | 0.95 (0.89, 1.03) | 0.201 |  | 1.15 (0.99, 1.32) | 0.062 |
| Waist circumference tertiles |  |  |  |  |  |  |  |  |
| T1 [0.51, 0.78] | *Ref* | |  | *Ref* | |  | *Ref* | |
| T2 [0.78, 0.86] | 0.89 (0.77, 1.03) | 0.115 |  | 1.09 (0.92, 1.30) | 0.328 |  | 1.31 (1.06, 1.61) | 0.012 |
| T3 [0.87, 1.17] | 0.72 (0.62, 0.83) | <0.001 |  | 0.97 (0.81, 1.17) | 0.769 |  | 1.42 (1.07, 1.88) | 0.015 |
| WHR |  |  |  |  |  |  |  |  |
| Per *SD* increase | 1.09 (1.03, 1.16) | 0.004 |  | 1.09 (1.02, 1.18) | 0.014 |  | 1.24 (1.13, 1.36) | <0.001 |
| WHR tertiles |  |  |  |  |  |  |  |  |
| T1 [0.53, 0.87] | *Ref* | |  | *Ref* | |  | *Ref* | |
| T2 [0.87, 0.93] | 1.04 (0.89, 1.20) | 0.639 |  | 1.14 (0.95, 1.36) | 0.162 |  | 1.34 (1.10, 1.63) | 0.003 |
| T3 [0.93, 1.68] | 1.23 (1.06, 1.43) | 0.006 |  | 1.30 (1.09, 1.55) | 0.004 |  | 1.74 (1.39, 2.17) | <0.001 |
| WHtR |  |  |  |  |  |  |  |  |
| Per *SD* increase | 1.02 (0.96, 1.08) | 0.501 |  | 1.01 (0.94, 1.09) | 0.763 |  | 1.51 (1.30, 1.75) | <0.001 |
| WHtR tertiles |  |  |  |  |  |  |  |  |
| T1 [0.34, 0.50] | *Ref* | |  | *Ref* | |  | *Ref* | |
| T2 [0.50 0.56] | 0.88 (0.76, 1.02) | 0.096 |  | 1.05 (0.88, 1.26) | 0.561 |  | 1.37 (1.11, 1.69) | 0.003 |
| T3 [0.56, 0.76] | 1.07 (0.92, 1.23) | 0.398 |  | 1.11 (0.93, 1.33) | 0.253 |  | 2.06 (1.55, 2.73) | <0.001 |
| ABSI*100 |  |  |  |  |  |  |  |  |
| Per *SD* increase | 1.42 (1.33, 1.52) | <0.001 |  | 1.17 (1.08, 1.26) | <0.001 |  | 1.17 (1.08, 1.26) | <0.001 |
| ABSI*100 tertiles |  |  |  |  |  |  |  |  |
| T1 [4.63, 7.89] | *Ref* | |  | *Ref* | |  | *Ref* | |
| T2 [7.89, 8.27] | 1.21 (1.04, 1.41) | 0.012 |  | 1.01 (0.84, 1.21) | 0.916 |  | 1.00 (0.83, 1.20) | 0.989 |
| T3 [8.27, 10.71] | 2.08 (1.79, 2.41) | <0.001 |  | 1.35 (1.13, 1.62) | 0.001 |  | 1.35 (1.12, 1.62) | 0.002 |
| BRI |  |  |  |  |  |  |  |  |
| Per *SD* increase | 1.02 (0.96, 1.09) | 0.464 |  | 1.01 (0.93, 1.08) | 0.856 |  | 1.50 (1.29, 1.74) | <0.001 |
| BRI tertiles |  |  |  |  |  |  |  |  |
| T1 [0.85, 3.39] | *Ref* | |  | *Ref* | |  | *Ref* | |
| T2 [3.39, 4.47] | 0.88 (0.76, 1.02) | 0.096 |  | 1.05 (0.88, 1.26) | 0.561 |  | 1.37 (1.11, 1.69) | 0.003 |
| T3 [4.47, 9.64] | 1.07 (0.92, 1.23) | 0.398 |  | 1.11 (0.93, 1.33) | 0.253 |  | 2.06 (1.55, 2.73) | <0.001 |
| Abbreviations:BMI, body mass index; WC, waist circumference; WHR, waist–hip ratio; WHtR, waist–height ratio; ABSI, a body shape index; BRI, body round index; baPWV, brachial-ankle pulse wave velocity; *Ref,* reference; *OR*, odds ratio; CI, confidence interval; *SD*, standard deviation. ModelⅠadjusted for age, sex, MAP and HR. ModelⅡadjusted for age, sex, MAP, HR, BMI, smoking and drinking status, DM, homocysteine, HDL-C, ALT and eGFR. | | | | | | | | |

**Supplemenary Figure**

**
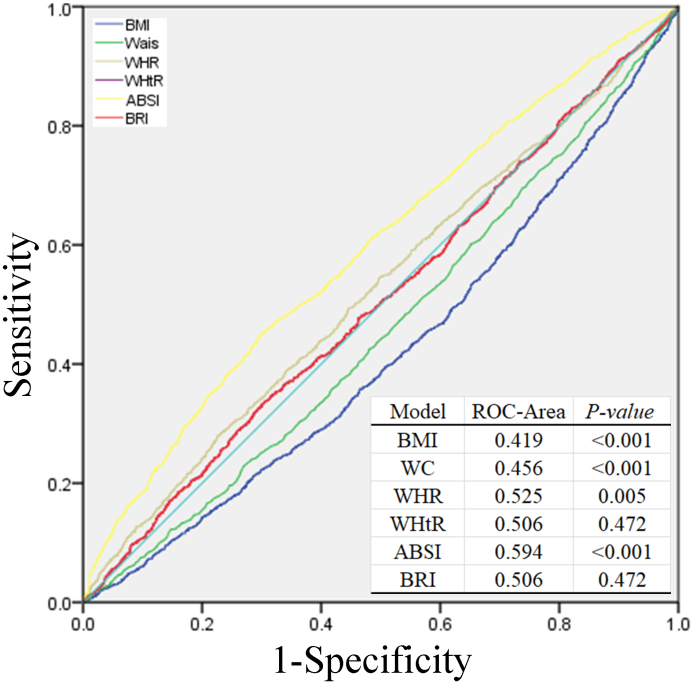
**

**Supplemenary Figure 1.** Receiver operating characteristic curve (ROC) analyses for the prediction of arterial stiffness based on baPWV values.

Abbreviations: BMI, body mass index; WC, waist circumference; WHR, waist–hip ratio; WHtR, waist–height ratio; ABSI, a body shape index; BRI, body round index.
